# Palmitic and linoleic acids induce ER stress and apoptosis in hepatoma cells

**DOI:** 10.1186/1476-511X-11-1

**Published:** 2012-01-05

**Authors:** Yong Zhang, Rongliang Xue, Zhenni Zhang, Xia Yang, Hongyang Shi

**Affiliations:** 1 Anesthesia Department, the Second Affiliated Hospital of Xi'an Jiaotong University, No. 157, West 5th Road, Xi'an, Shaanxi Province-710004, China; 2 Department of Respiration, the Second Affiliated Hospital of Xi'an Jiaotong University, No. 157, West 5th Road, Xi'an, Shaanxi Province-710004, China

**Keywords:** Nonalcoholic fatty liver disease, Palmitic, Linoleic, Endoplasmic Reticulum Stress, BAPTA-AM; Thapsigargin

## Abstract

**Objectives:**

Hepatic inflammation and degeneration induced by lipid depositions may be the major cause of nonalcoholic fatty liver disease. In this study, we tried to investigate the effects of saturated and unsaturated fatty acids on hepatoma cell apoptosis.

**Methods:**

H4IIE liver cells were treated with palmitic acid, linoleic acid, or both with or without the calcium-specific chelator BAPTA-AM after which the expression of proteins associated with endoplasmic reticulum (ER) stress, apoptosis, caspase-3 levels, and calcium flux were measured.

**Results:**

Palmitic or linoleic acid (250 μM) induced H4IIE cell apoptosis, which required calcium flux but not caspase-3. Apoptosis was not observed when cells were co-treated with linoleic acid (125 μM) and palmitic acid (250 μM). Importantly, the release of cytochrome C from mitochondria into cytoplasm during cell apoptosis was specifically detected only when linoleic acid (125 μM), but not palmitic acid (250 μM), was added to the cells. Depletion of intracellular calcium flux by the calcium-specific chelator, BAPTA-AM, abolished linoleic acid-induced apoptosis. Moreover, in the presence of BAPTA-AM, expression of the unfolded protein response (UPR)-associated genes, CHOP, GRP78, and GRP94, was induced by linoleic acid, but not palmitic acid.

**Conclusions:**

The results suggest that linoleic acid promotes cell apoptosis through the release of cytochrome C, only if the intracellular calcium flux is unperturbed and intact. These results confirm that ER stress contributes to fatty acid-induced liver cell apoptosis.

## 1. Background

Nonalcoholic fatty liver disease (NAFLD) is a multifactorial disease [1] that manifests as symptoms, ranging from mild steatosis, to nonalcoholic steatohepatitis and cirrhosis in the liver. Although NAFLD affects millions of people worldwide, its etiology remains unclear. However, hepatic inflammation and degeneration induced by the deposition of lipid droplets in the organ has been established as one of the major causes of the disease [1-3]. In particular, certain saturated fatty acids, such as palmitic acid, can induce endoplasmic reticulum (ER) stress and apoptosis in rat and human liver cell lines [4-9], leading to inflammation and/or degeneration in the liver. This hypothesis was further supported by the observation that ER stress and apoptosis could be induced by palmitic acid in both primary cells and cell lines derived from mice and rats [10,11].

Overconsumption of fat-containing foods is one of the major contributors to obesity and NAFLD. Dietary fatty acids in the liver may provoke ER stress and apoptosis in liver cells, but controversy remains over the differential effects exerted by saturated and unsaturated fatty acids with respect to liver damage. For example, Vecchini et al. [12] demonstrated hepatoma cell apoptosis in response to unsaturated α-linolenic acid, and a later study indicated that a polyunsaturated fatty acids can augment the effects of a saturated fatty acids on inducing ER stress and apoptosis in liver cells [8]. However, other evidence strongly suggested that unsaturated fatty acids can protect against saturated fatty acid-mediated ER stress and apoptosis in the kidney [13], fibroblasts [14], liver cells [7], and macrophages [15].

The biological basis for why saturated fatty acids are often lipotoxic whereas unsaturated fatty acids appear to be protective is poorly understood and requires clarification [16]. Our previous studies show that unsaturated fatty acid alpha-linoleic acid could reduced ER stress-mediated apoptosis of saturated fatty acids palmitic acid and stearic acid lipotoxicities in primary rat liver cells [17,18]. Whether this phenomenon also exists in liver tumor cells is unknown.

A previous study by Wei et al. [7] showed that the exposure of rat hepatoma H4IIE cells to saturated fatty acids (e.g., palmitic acid and, to a lesser extent, stearic acid) reduced thapsigargin-sensitive calcium stores and increased the expression of biochemical markers of ER stress, leading to cell death. However, co-incubation of the cells with oleic acid--and, to a lesser extent, linoleic--prevented the effects induced by palmitic acid. Linoleic acid, a polyunsaturated essential fatty acid, is abundant in diets that have been associated with the pathogenesis of NAFLD. The objective of this study was to determine the underlying mechanism of the interaction between linoleic acid and palmitic acid on ER stress and apoptosis in rat hepatoma H4IIE liver cells.

## 2. Materials and Methods

### 2.1. Materials and reagents

Palmitic and linoleic acids, thapsigargin, and other reagents were purchased from Sigma-Aldrich (St. Louis, MO) unless otherwise specified. The calcium-specific chelator BAPTA-AM (1,2-bis-(*O*-aminopenoxy)-ethane-*N, N, N', N'*-tetraacetic acid tetraacetoxymethyl ester) was obtained from Biomol International Lp/Enzo Life Sciences (Plymouth Meetings, PA).

### 2.2. Cell culture

Rat hepatoma H4IIE liver cells (American Type Culture collection, Manassas, VA) were cultured in Dulbecco's Modified Eagle's Medium supplemented with 10% fetal bovine serum, penicillin, and streptomycin sulfate. This medium was defined as the control medium (LG), and was prepared with or without 125 μM or 250 μM palmitic acid or linoleic acid as previously described [8]. Briefly, fatty acids were complexed to bovine serum albumin at a 2:1 molar ratio by treating a 20 mM solution of fatty acid in 0.01 M NaOH at 70°C for 30 min. Addition of 1 N NaOH was used to promote solubilization. Fatty acid soaps were then complexed with fatty acid-free BSA in phosphate-buffered saline to achieve the appropriate 2:1 (fatty acid-to-albumin) molar ratio. The fatty acid concentrations selected (125 and 250 μM) were based on those reported by Wei et al. [7]. The cells were treated for 16 hours in each condition. In calcium flux study, the cells were treated with indicated acids in the absence or presence of 10 μM calcium-specific chelator BAPTA-AM for 16 hours.

### 2.3. RNA isolation and real-time RT-PCR

Total RNA was extracted with TRIzol reagent (Invitrogen, Carlsgbad, CA) according to the manufacturer's instructions. A 5-μl sample of DNase-treated RNA was used for reverse transcription with Superscript II RNaseH and random hexamers. Real-time polymerase chain reaction amplification of the transcribed cDNA was performed with the IQ-SYBR green master mix (BioRad, Hercules, CA). The sequences of the six primer sets were as previously reported (Supplemental Table One [9]).

The PCR efficiency was between 90% and 105% for all primer and probe sets and linear over five orders of magnitude. The specificity of products generated for each set of primers was examined for each fragment by melting curve and gel electrophoresis. Reactions were run in triplicate, and data were calculated as the change in cycle threshold (ΔCT) for the target gene relative to the ΔCT for β_2_-microglobulin according to the procedures of Muller et al. [19].

### 2.4. Western blot analysis

Western blot analysis was performed as described previously [9]. Primary antibodies used were against GRP78 (Stressgen, Victoria, BC, Canada), GRP94 (Santa Cruz Biotechnology, Santa Cruz, CA), CHOP (Santa Cruz Biotechnology, Santa Cruz, CA), and ß-actin (Sigma Chemical Company, St. Louis, MO). Expression levels of the respective proteins were determined with horseradish peroxidase-conjugated secondary antibodies (GE Healthcare, Piscataway, NJ) and enhanced chemiluminescence reagent (Pierce, Rockford, IL). Band densities were quantified with a UVP BioImaging system (Upland, CA) and normalized against ß-actin.

### 2.5. Measuring caspase-3 activity

Caspase-3 activity was measured according to a colorimetric assay that measures the extent of cleavage of a caspase-specific peptide derivatized with p-nitroalanine, such that upon proteolysis, the chromophore is released and the concentration is measured spectrophotometrically. The assay was performed according to the manufacturer's instructions (R&D Systems, Inc.). Briefly, cells stained with Trypan blue were counted by hemocytometer, and approximately 10^6 ^cells were used in each assay. Cells were pelleted (250 × g for 10 minutes), the supernatants were discarded, and the pellets were lysed. Protein content within lysates was estimated using the BCA Protein Assay (Pierce, Rockford, IL), and enzymatic reactions for caspase activity were carried out in 96-well flat bottom microplates. Following incubation with the Caspase-3 colorimetric substrate (DEVD-pNA), the extent of proteolysis was measured from the colorimetric intensity as read by a microplate reader at 405 nm. Control reactions were run without cell lysate and without substrate.

### 2.6. Analysis of apoptosis and cytochrome C assay

Cells were harvested in PBS and collected by centrifugation as described previously [7], and the cytochrome C content in the post-mitochondrial supernatant was determined by ELISA using a kit (Invitrogen, Carlsbad, CA). Cell death was evaluated with the Cell Death Detection ELISA kit (Roche Diagnostics, Penzberg, Germany), which uses mouse monoclonal antibodies directed against DNA and histones.

### 2.7. Measurement of calcium flux

Changes in calcium flux were determined with the Fluo-4 NW calcium assay kit (Molecular Probes, Eugene, OR). Briefly, cells were loaded with Fluo-4 NW in the presence of probenecid (4-dipropylamino-sulfonyl benzoic acid), and the fluorescence (494 nm excitation, 516 nm emission) was measured over the time points indicated.

### 2.8. Statistical Analysis

Means and standard deviations were calculated for each group. Comparisons were performed by ANOVA with post-hoc comparison adjusted by the Bonferroni method. Data were analyzed with SAS 9.0 (SAS Institute Inc., Cary, NC). Values of *P *< 0.05 were considered statistically significant.

## 3. Results

### 3.1. Effects of fatty acids on H4IIE cell apoptosis, caspase 3 activity and calcium level

**Figure 1:**
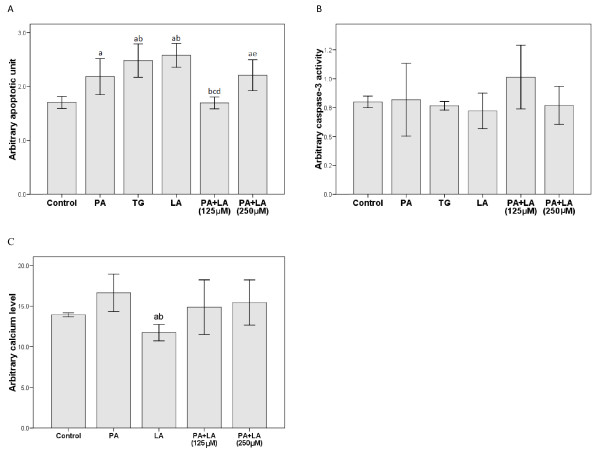
**Effects of palmitic and linoleic acids on apoptosis, caspase-3 activity and calcium flux in H4IIE liver cells**. Rat liver cells were treated with thapsigargin (TG), palmitic acid (PA, 250 μM), linoleic acid (LA, 250 μM) alone or the combination of PA (250 μM) and LA (125 or 250 μM) for 16 hours. The cells were treated with phosphate buffered saline (PBS) as Control group. (A) Apoptotic index was measured by determining the cytoplasmic histone-associated DNA fragments with an ELISA assay. (B) The caspase-3 level was detected according to a colorimetric assay that measures the extent of cleavage of a caspase-specific peptide derivatized with p-nitroalanine. (C) The calcium level was determined with the Fluo-4 NW calcium assay kit. a: P < 0.05 compared to Control; b: P < 0.05 compared to PA; c: P < 0.05 compared to TG; d: P < 0.05 compared to LA; e: P < 0.05 compared to PA+LA (125uM) after Bonferroni adjustment. *n *= 6 in each group.

The apoptotic response of rat liver H4IIE cells to the fatty acid treatments was first confirmed by measurement of cytoplasmic histone-associated DNA fragments, as shown in Figure 1A. Treatment of cells for 16 h with thapsigargin (2 μM), palmitic acid (250 μM), or linoleic acid (250 μM) significantly increased cell death compared with the untreated control cells (*P *< 0.05). Notably, linoleic acid induced significantly greater apoptosis than the same concentration of palmitic acid at the same time point (*P *< 0.05). Interestingly, the combination of PA and 125 μM LA but not 250 μM LA significantly decreased apoptosis in comparison to PA treatment alone. However, no significant changes in caspase-3 activity were observed in response to fatty acid treatments (Figure 1B).

Because caspase-3 activity remained unaltered in response to fatty acid treatment, ER calcium flux was analyzed to determine if it was involved in the apoptosis observed in Figure 1A. As shown in Figure 1C, increased calcium flux was observed in cells treated with palmitic acid (250 μM) for 16 h. However, linoleic acid (250 μM) decreased calcium flux at 16 h whereas no differences were observed with palmitic and linoleic acid cotreatments (Figure 1C).

### 3.2. Effects of palmitic and linoleic acids on UPR-associated gene expression

**Figure 2:**
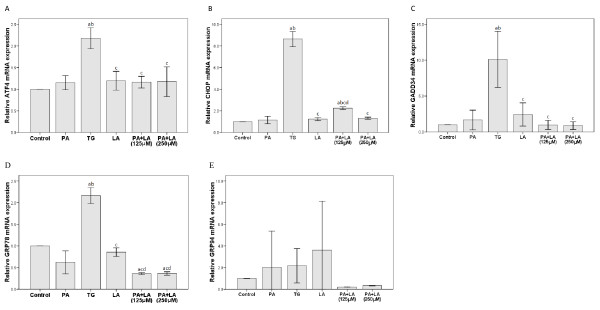
**Effects of palmitic and linoleic acids on the expression of UPR-associated genes in mRNA level**. The tumor cells were treated with TG, PA(250 μM), LA (250 μM) alone or PA+LA (125 μM or 250 μM) for 16 hr. The cells were treated with PBS as Control group. The levels of the respective mRNAs for (A) ATF4, (B) CHOP, (C) GADD34, (D) GRP78, and (E) GRP94 were determined by real-time RT-PCR relative to β2-microglobulin. a: P < 0.05 compared to Control; b: P < 0.05 compared to PA; c: P < 0.05 compared to TG; d: P < 0.05 compared to LA after Bonferroni adjustment. *n *= 3 in each group.

**Figure 3:**
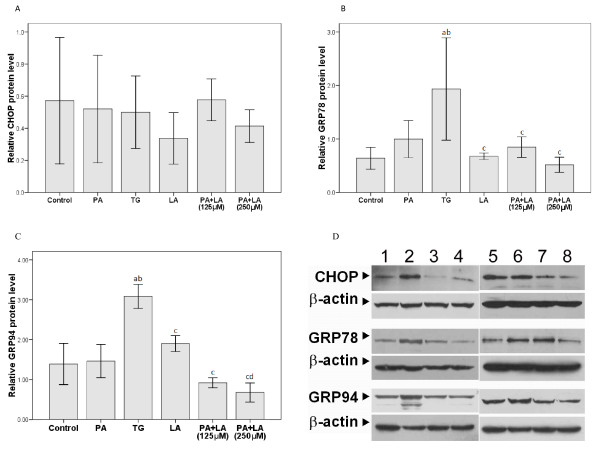
**Effects of palmitic and linoleic acids on the expression of CHOP, GRP78, and GRP94 in protein level**. The Rat liver tumor cells were treated with TG, PA(250 μM), LA (250 μM) alone or PA+LA (125 μM or 250 μM) for 16 hr. The cells were treated with PBS as Control group. The levels of the respective proteins were determined by Western blot and normalized to the level of β-actin (*n *= 3 in each group). The relative expression of protein (A) CHOP, (B) GRP78 and (C) GRP94 were summarized and representative western blot photos were presented in (D). a: P < 0.05 compared to Control; b: P < 0.05 compared to PA; c: P < 0.05 compared to TG after Bonferroni adjustment. In (D), lane 1 and 5 were loaded with Control, lane 2 for TG, lane 3 and 6 for PA, lane 4 for LA, lane 7 for PA+LA (125 μM) and lane 8 for PA+LA (250 μM).

The unfolded protein response (UPR) is the main pathway related to ER stress [20]. Therefore, the expression of genes involved in the UPR, ATF4, CHOP, GADD34, GRP78, and GRP94, was assessed after fatty acid treatment for 16 h (Figure 2 and 3). Significantly increased ATF4, CHOP, GADD34, and GRP78 mRNA expression was observed upon treatment with thapsigargin (2 μM); no changes in GRP94 expression were observed (Figure 2E). Changes in the H4IIE cells were observed upon cotreatment with palmitic and linoleic acid. Significantly increased CHOP mRNA expression was observed upon cotreatment (125 μM) whereas GRP78 mRNA expression was significantly decreased with cotreatment at both concentrations (Figure 2B and 2D). However, these changes in the H4IIE cells were not observed at the protein level for either of these genes (Figure 3A and 3B). Increased GRP78 and GRP94 protein expression (Figure 3B and 3C, respectively) were also observed upon thapsigargin treatment; however, no changes in CHOP protein expression were observed (Figure 3A). In contrast, treatment of H4IIE cells with 250 μM palmitic or linoleic acid alone failed to alter the mRNA or protein expression of any of these genes. A representative Western blot for each protein after the indicated treatments is shown in Figure 3D.

### 3.3. Role of calcium in fatty acid-induced liver cell apoptosis and ER stress

**Figure 4:**
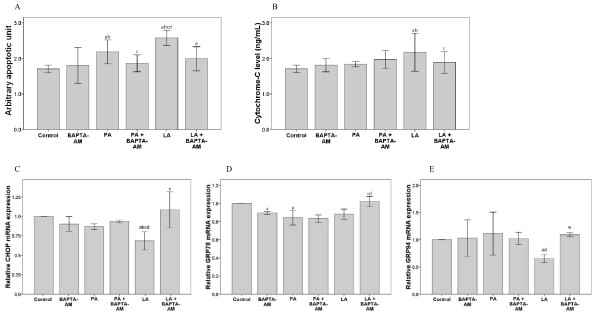
**Impact of calcium flux on cell death, cytochrome C release, and expression of CHOP, GRP78, and GRP94 genes in cells treated with palmitic acid or linoleic acid in the absence and presence of 10 μM calcium-specific chelator BAPTA-AM**. H4IIE liver cells were treated with PA or LA (125 μM) in the absence or presence of 10 μM calcium-specific chelator BAPTA-AM for 16 hours (n = 3 in each group).

The role of intracellular calcium in fatty acid-induced H4IIE cell apoptosis and ER stress was analyzed using the highly specific calcium chelator, BAPTA-AM (10 μM; Figure 4). As shown in Figure 4A, BAPTA-AM treatment alone for 16 h did not alter H4IIE cell apoptosis. However, it did significantly reduce the cell death induced by both palmitic and linoleic acids (Figure 4A).

Cytochrome C levels were also determined after treatment with fatty acids; palmitic acid (250 μM) did not affect its level in the absence or presence of BAPTA-AM (Figure 4B). However, cytochrome C levels were increased significantly upon treatment with linoleic acid (125 μM), which was reversed with BAPTA-AM (Figure 4B; *P *< 0.05). Thus, saturated palmitic acid and unsaturated linoleic acid may mediate their pathologic effects in the liver through different calcium-dependent apoptotic pathways (i.e., linoleic acid, but not palmitic acid, requires calcium flux and the release of cytochrome C to induce cell apoptosis).

Consistent with this hypothesis, cells treated with BAPTA-AM and 125 μM linoleic had increased CHOP, GRP78, and GRP94 mRNA expression as compared to those treated with linoleic acid alone; this was not observed for palmitic acid (Figure 4C-E). The mRNA expression levels for CHOP and GRP94 were lower in cells treated with linoleic rather than palmitic acid (Figure 4C and 4E). These results again indicate that calcium is required for the cytochrome C-dependent apoptosis promoted by the expression of certain genes important in UPR.

## 4. Discussion

The objective of this study was to examine the hypothesis that palmitic acid induces ER stress and cell death in hepatoma cells, which is inhibited by linoleic acid. Thus, the effects of these fatty acids, alone and in combination, on apoptosis, caspase-3 activity, cytochrome C levels, ER stress, calcium flux changes, and mRNA and protein expression of ER stress-associated genes were examined in rat hepatoma H4IIE cells.

Linoleic acid induced significantly more apoptosis than palmitic acid, which is consistent with an earlier report in smooth muscle cells in which palmitic and linoleic acids were assessed at physiological mixtures (300-900 μM) over 24-72 h [4]. Because plasma FFA and TG in rodents reach approximately 500 μM, future analyses of the effects of linoleic and palmitic acid will include higher concentrations.

Both palmitic and linoleic acid evoked a significant elevation of calcium flux (ER stress) at 16 h, which is in relatively good agreement with one previous report [7]). Fatty acid treatment had no effect on caspase-3 activity, indicating that apoptosis of H4IIE cells induced by either fatty acid was probably not mediated through this pathway. These results contrasted with previous reports, which demonstrated that saturated fatty acids increased caspase-3 and caspase-9 activity [8], and that DCP-LA, a derivative of linoleic acid, can protect neurons from oxidative stress-induced apoptosis by inhibiting caspase-3 and -9 activation [21]. Further experiments on the role of other caspases, such as caspase-12 [20,22], will be pursued. The apoptotic pathway activated by palmitic and linoleic acid is therefore distinct from that inhibited by metformin, the antidiabetic drug reported to inhibit palmitate-induced apoptosis via activation of caspase-3 [5]. Like unsaturated fatty acids, metformin also blocked the induction of ER stress proteins, and may protect hepatocytes from death induced by saturated fatty acids [5].

Previous investigators have reported that treating a range of cell-types with unsaturated fatty acids protected the cells against increased apoptosis and lipotoxicity caused by saturated fatty acids, including palmitic acid [10,13,14,16,21,23]. In contrast, we observed that apoptosis was greater in cells treated with 250 μM linoleic acid alone than with the same concentration of palmitic acid. Furthermore, a protective effect against palmitic acid-induced apoptosis by linoleic acid was observed only at 125 μM, but not 250 μM, which is inconsistent from our previous reports in primary liver cells [17,18]. Use of a hepatoma cell line in this study versus primary rat liver cells in previous studies [17,18] may account for these discrepancies.

In the present study, caspase-3 was not involved in mediating fatty acid-induced apoptosis, and direct measurements of calcium flux indicated no involvement in apoptosis. However, the addition of the calcium specific chelator, BAPTA-AM, prevented apoptosis induced by palmitic acid alone, as well as palmitic acid plus 250 μM linoleic acid, suggesting that calcium does indeed play a role in the apoptotic response. More importantly, cytochrome C release was detected only when unsaturated linoleic acid (125 μM), but not the saturated palmitic acid (250 μM), was added to the cells, and depletion of the intracellular calcium pool abolished this linoleic acid-mediated effect. The effects on cytochrome C release were again in sharp contrast to previous reports in which increased cytochrome C in post-mitochondrial supernatants were detected after treatment with palmitic acid [7]. Thus, liver damage may be caused by different fatty acids through alternate apoptotic pathways, and whether or not linoleic acid can protect the liver from palmitic acid-induced apoptosis depend on the balance of influences exerted by the different apoptotic/ER stress pathways. This may resolve the controversies stemming from the observation that dietary saturated and unsaturated fatty acids provoke ER stress and apoptosis differently in liver cells [7,8,12-14].

Monitoring changes in the mRNA levels of UPR-related genes provided a direct way of measuring the effects of palmitic and linoleic acid on ER stress. Wei et al. [7] reported increased expression of the UPR-associated genes, ATF4, CHOP, GADD34, and GRP78 after incubation with 250 μM palmitic or unsaturated (oleic) acid after only 6 h. Wei et al. [7] also reported that 125 μM oleic acid (an unsaturated FA) reversed the apoptotic and ER stress effects induced by 250 μM palmitic acid, in direct agreement with the data presented here using linoleic acid. Ishiyama et al. [15] reported that oleic acid and linoleic acid (at 200 μM) suppressed the upregulation of the oxidized LDL receptor-1 by palmitic acid (200 μM) or by thapsigargin (2 μM), reducing the uptake of oxidized LDL by macrophages and alleviating ER stress. Thus, unsaturated FA can ameliorate the apoptotic and ER stress effects of palmitic acid or other saturated FA.

Studies investigating the effects of FA upon apoptosis and ER stress in cells are often inconsistent; some inconsistencies are due to different incubation times, FA concentration, total dosage and exposure time, and cell lines. For example, Karaskov et al. [23] reported that 1 mM palmitic acid upregulated some but not all UPR related genes in a pancreatic ß-cell line, where levels of ATF4 and CHOP increased, but GRP78 levels remained unchanged, which are in contrast to our results. The present study also analyzed another important UPR-related gene, GRP94 [20], for its FA-induced gene expression profile. The gene expression induced by either FA was mild, if not suppressed, and the elevated levels were similar. Simultaneous treatment with both FA revealed the induction and suppression of the CHOP and GRP94 gene expression levels, respectively, which reached significance when liver cells were co-incubated with 125 μM of linoleic acid and 250 μM palmitic acid; further increasing the linoleic acid concentration to 250 μM reversed the suppressive effects of 125 μM linoleic acid on mRNA expression. However, similar changes in protein concentration were not observed. The concentration-dependence of the linoleic acid-mediated suppression may reflect other activities for this FA in addition to the anti-lipotoxic role described here, such as generating mutagenic metabolites capable of damaging DNA in response to oxidative stress [14].

Overexpression of GRP78 can provide resistance to cells against apoptosis induced by thapsigargin [24], and so it is significant that cells treated with thapsigargin contained higher levels of expressed GRP78 after 16 h in the present study. Increased GRP78 expression may help cells adapt against ER stress induced by thapsigargin. GRP78 has even been reported to protect Mouse MIN6 pancreatic ß-cells from palmitate-induced apoptosis [25].

In summary, the effects of palmitic and linoleic fatty acids on H4IIE cell apoptosis and ER stress were investigated. The results strongly suggest that both palmitic and linoleic acid required the flux of calcium to mediate ER stress. Furthermore, cytochrome C, but not caspase-3, was selectively involved in FA-induced cell apoptosis. Among the genes that are important for UPR, it is likely that CHOP and GRP94 play a role in mediating the physiologic phenomenon. In contrast with previous studies [8,9], caspase-3 seemed to play no role in FA-induced liver apoptosis, but this and other conclusions drawn from this study warrant further investigation.

## Competing interests

The authors declare that they have no competing interests.

## Authors' contributions

YZ conceived, designed and coordinated the work, as well as prepared the manuscript. RX was involved in the co-design of the work as well as the draft of the manuscript. ZZ, HS and XY carried out analytical work and contributed in drafting the manuscript. All authors read and approved the final manuscript. 
